# Orexins/Hypocretins: Key Regulators of Energy Homeostasis

**DOI:** 10.3389/fendo.2019.00830

**Published:** 2019-12-10

**Authors:** Edward Milbank, Miguel López

**Affiliations:** ^1^Department of Physiology, CIMUS, Instituto de Investigación Sanitaria, Santiago de Compostela, Spain; ^2^CIBER Fisiopatología de la Obesidad y Nutrición (CIBERobn), Santiago de Compostela, Spain

**Keywords:** orexins/hypocretins, hypothalamus, food intake, thermogenesis, brown adipose tissue

## Abstract

Originally described to be involved in feeding regulation, orexins/hypocretins are now also considered as major regulatory actors of numerous biological processes, such as pain, sleep, cardiovascular function, neuroendocrine regulation, and energy expenditure. Therefore, they constitute one of the most pleiotropic families of hypothalamic neuropeptides. Although their orexigenic effect is well documented, orexins/hypocretins also exert central effects on energy expenditure, notably on the brown adipose tissue (BAT) thermogenesis. A better comprehension of the underlying mechanisms and potential interactions with other hypothalamic molecular pathways involved in the modulation of food intake and thermogenesis, such as AMP-activated protein kinase (AMPK) and endoplasmic reticulum (ER) stress, is essential to determine the exact implication and pathophysiological relevance of orexins/hypocretins on the control of energy balance. Here, we will review the actions of orexins on energy balance, with special focus on feeding and brown fat function.

## Introduction

Localized below the thalamus on the ventral face of the diencephalon, the hypothalamus is a master center for the integration of multiple signals and participates in the regulation of numerous homeostatic functions; the regulation of energy balance and endocrine axes being the most important ones (1–5). The hypothalamus is composed of anatomically distinct nuclei emitting numerous axonal projections interconnecting one region to another developing a complex neuronal circuit. One of the most important hypothalamic nuclei involved in the feeding regulation is the arcuate nucleus (ARC), which is composed by two main neuronal populations: (i) orexigenic (feeding-promoting) neurons co-expressing neuropeptide Y (NPY) and agouti-related protein (AgRP) and (ii) anorexigenic (feeding-inhibiting) ones co-expressing cocaine- and amphetamine- related transcript (CART) and pro-opiomelanocortin (POMC; the precursor of the alpha-melanocyte stimulating hormone, α-MSH). Following their integration by the ARC, the peripheral signals are transmitted by neuronal projections to other hypothalamic areas such as the dorsomedial nucleus (DMH), the paraventricular nucleus (PVH) and the lateral hypothalamic area (LHA). The ventromedial nucleus of the hypothalamus (VMH; localized above and laterally from the ARC on both sides of the third ventricle) is also receiving secondary neuronal information originating from the ARC. VMH neurons subsequently communicate, through neuronal projections, with other hypothalamic areas, such as DMH, LHA and ARC, as well as other cerebral regions, such as the dorsal motor nucleus of the vagus (DMV), the nucleus tractus solitarius (NTS), the raphe pallidus (RPa) and the inferior olive (IO) (1–5).

The importance of the hypothalamus in the modulation of autonomic and endocrine functions was described for the first time more than 70 years ago (1, 5–11). A precise fine tuning of these functions is crucial for numerous physiological processes, such as drinking and feeding, thermoregulation, neuroendocrine control, reproduction/lactation, sleep-wake cycle, and cardiovascular function. Even if the role of LHA in the above described functions has been fully identified (1, 5–11), the underlying molecular mechanisms remained poorly studied until the latter part of the twentieth century, when the physiological role of some LHA-expressed neuropeptides was discovered, among then orexins/hypocretins.

## The Discovery of Orexins/Hypocretins

Melanin-concentrating hormone (MCH) was the first discovered orexigenic peptide located specifically in the LHA (12–14). Other feeding related neuropeptides, such as galanin (GAL) (15), dynorphin (DYN) (16), and cocaine- and amphetamine-regulated transcript peptides (CARTps) (17) have later also been described to be expressed in the LHA. Nevertheless, the most relevant finding has been the discovery in 1998 of a new family of neuropeptides: the *orexins/hypocretins*. *Sutcliffe* and colleagues, using the directional tag polymerase chain reaction subtraction, were able to identify a novel mRNA, which expression was limited to the LHA. This mRNA was described to encode a 130-amino acid secretory protein composed of a proteolytic site engendering two C-terminally amidated peptides. Interestingly, significant homologies were observed between one of these generated peptides and the gut secretin peptide family. Therefore, due to their hypothalamic origins, they were named *hypocretins* (Hcrt1 and Hcrt2) (18). In parallel, *Yanagisawa* and colleagues identified two peptides using intracellular calcium influx in high orphan G-protein-coupled receptor expressing cells. They were able to determine that the two neuropeptides were originating from a common proteolytic processed LHA precursor, and, considering its hypothalamic location, they have suggested its potential role in feeding behavior. In line with this assessment, intracerebroventricular (ICV) administrations of these peptides in non-fasted rats stimulated food intake in a dose- and time-dependent fashion. Therefore, considering their orexigenic activities, these neuropeptides were named *orexins* (OX-A and OX-B) (19). Nowadays, orexins are known to participate widely in the regulation of numerous biological processes, such as sleep, energy expenditure, pain, cardiovascular function, and neuroendocrine regulation, making them one of the most pleiotropic families of hypothalamic neuropeptides (9, 20–26). In this regard, it is important to take into account that although most of the first studies on orexins were based on their administration through different routes and doses that were likely outside of a physiological range (see below), further studies using genetic modified animals (see below) have strengthen the pleiotropic role of this neuropeptide system.

Two orexin/hypocretin receptors were identified: the orexin/hypocretin 1 receptor (OX1R/Hcrtr1), having a highest affinity for OX-A, and the orexin/hypocretin 2 receptor (OX2R/Hcrtr2), having similar elevated affinity for both OX-A and OX-B (19). High levels of OXRs mRNA and proteins are encountered in the central nervous system. Molecular and immunohistochemistry studies have shown that OX1R neurons were highly expressed in rat hypothalamus, with a main distribution in the periventricular (PeN), PVH (magno- and parvocellular divisions), supraoptic (SON), ARC, VMH, DMH, and tuberomammillary (TMN) nuclei, as well as in the LHA (27–30). Interestingly, OX1R and neuropeptides participating in the endocrine regulation are sharing the same central distribution. As an example, in the SON and in magnocellular neurons of the PVH, OX1R is expressed in arginine vasopressin (ADH) and oxytocin (OT) neurons. Similar observations were performed in the suprachiasmatic nucleus (SCN) with an expression of OX1R (i) in ADH and vasoactive intestinal polypeptide (VIP) neurons, (ii) in the PeN with OX1R-somatostatin (SST) co-expressing neurons, and (iii) and in the parvocellular part of PVH with OX1R colocalized in corticotropin-releasing hormone (CRH) neurons (30). Moreover, OX1R is also expressed in the main neuronal populations constituting the ARC: POMC neurons of the ventrolateral part and NPY neurons of the ventromedial part (30). Moreover, OX1R expression was also detected in other brain regions of the rat such as in the septal preoptic area (SPOA), in the medial preoptic area MPOA (27, 29, 31) and in gonadotropin-releasing hormone (GnRH) neurons (32). As OX1R, OX2R mRNAs is widely encountered within the brain with high levels in the ARC and LHA, in the medial parvocellular part of the PVH, in the premammillary nucleus (PMN) and TMN. Within the VMH, DMH, PeN, in the posterior hypothalamus and the preoptic area (POA), the detected levels of OX2R mRNAs were lower, reaching really weak levels in the SPOA and in the dorsal and lateral parts of the PVH. Notably, OX2R mRNAs were not detected in the magnocellular neurons of the PVH (27–29, 33). OXRs wide distribution all around the central nervous system confirm their pleiotropic role. In terms of signal transduction, the binding of orexins to OX1R or OX2R stimulates Gq or Gi subtypes, which subsequently induce the activation of phospholipase C (PLC), phospholipase A (PLA), phospholipase D (PLD) or adenylyl cyclase (AC), ultimately resulting in an increase in cytosolic Ca^2+^ and a downstream cascade response. In addition, OX-A binds OX1R and stimulates Ca^2+^ release by activating non-selective cation channels (NSCCs) (34). Finally, OX1R receptor activation also promotes the enhancement of the synthesis of the endocannabinoid 2-arachidonoylglycerol (2-AG) which is a master interplayer with OX-A in the regulation of energy homeostasis both at central and peripheral level (35, 36).

## Orexin/Hypocretins and Food Intake

The first physiological function attributed to the orexin system was the modulation of the feeding behavior. This was suggested following two major studies: **(i)** the hypothalamic expression of orexin precursor was increased during fasting (19, 37) and **(ii)** the pharmacological central administration of orexin in rats induced food intake (food consumption being dependent of the orexin injected dose) (19, 38–40). This feeding-promoting effect is not as robust as the one induced by NPY and AgRP, but it is considered as similar as the one initiated by MCH and GAL (38, 40–45). Although the precise effect of OX-B on feeding remains unclear, its orexigenic potential was described to be less potent than OX-A (38, 41–46), which could be explained by differences in their secondary structures: OX-A being maintained by two disulphide bonds conferring resistance to peptidase actions (20).

Interestingly, in rat models, both central OX-A and OX-B administrations induced orexigenic effects. Conversely, when injected peripherally (acute or chronic), none of the orexins induced feeding variations (41), except one study in pigs showing that the subcutaneous injection of OX-B stimulated food intake (47). Moreover, orexin actions have been described to follow circadian patterns, with a maximal effect at the beginning of the light phase and at the middle of the dark one (observed when animals are satiated) (40, 41). These differences could be explained by the circadian variations of the endogenous levels of OX-A, described to be maximal at the beginning of the dark phase and minimal at the beginning of the light one (48, 49). Interestingly, orexigenic effects of orexins were also observed in non-mammalian species, such as in the goldfish (*Carassius auratus*) (administrated centrally with human OX-A) (50), suggesting a possible evolutionary conservation. However, another study led on neonatal chicks did not reveal any feeding variations following a central administration of orexins (51).

Although most of these evidences have been obtained following administrations of orexins at doses that are likely higher than the physiological range, some studies have also revealed an implication of the orexin system in the physiological control of feeding. As an example, the central administration of anti-OX-A antibodies in rats under fasting conditions inhibited feeding in a dose-dependent manner (52). In the same line of findings, OX1R antagonist (SB-334867-A) administrated intraperitoneally decreased food intake in both fed and fasting conditions (39). Interestingly, the administration of OX-A at small doses in hypothalamic nuclei such as PVH and LHA of rodent models stimulates food intake (43, 53), while the selective OX antagonist SB-334867 suppresses it (39, 54). Due to the OX-A higher orexigenic potential compared to OX-B, these data implicate OX1R rather than OX2R in the modulation of feeding behavior (39, 55). These findings were confirmed using genetic modified mouse models, such as orexin knockout mice (*ox*^−/−^) (20) and orexin/ataxin-3 transgenic mice (in which orexin-containing neurons were abalated) (56), models that displayed hypophagic behaviors. Therefore, despite the unequivocal role of orexins in the modulation of other phycological processes, such as sleep (57–59), their physiological role in food intake control seems also clear.

**Figure 1:**
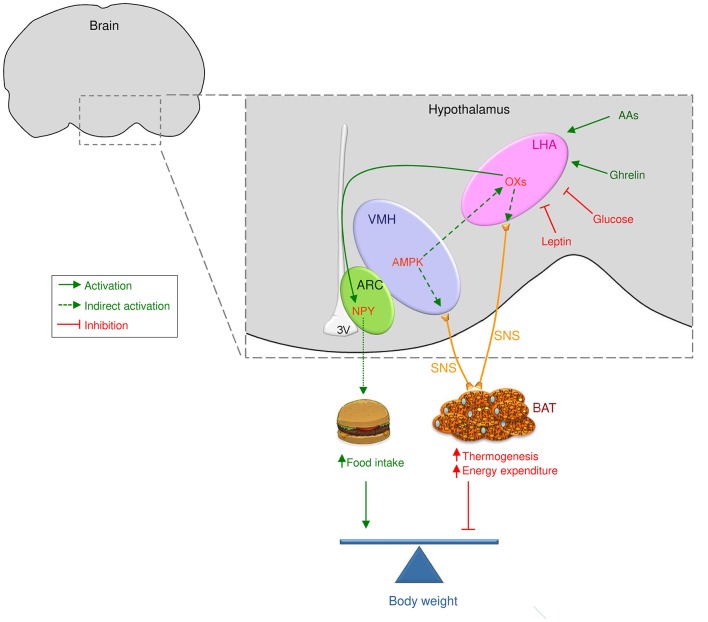
Central actions of orexins/hypocretins on food intake and BAT thermogenesis. Orexin/hypocretin neurons sense peripheral levels of metabolites, such as glucose and amino acids, and hormones, such as leptin and ghrelin, to control energy homeostasis. Therefore, orexin/hypocretin neurons in the lateral hypothalamic area (LHA) modulate food intake acting on neuropeptide Y (NPY) neurons in the arcuate nucleus of the hypothalamus (ARC). On the other hand, AMP-activated protein kinase (AMPK) in the ventromedial nucleus of the hypothalamus (VMH) impacts orexin neurons in the LHA to modulate brown adipose tissue (BAT) thermogenesis through the sympathetic nervous system (SNS, indicated by yellow lines). Whether OX may impact other central mechanism modulating thermogenesis, such as endoplasmic reticulum stress (ER stress) within the VMH will require further investigation.

In agreement with a physiological role of orexin system in feeding control, the orexigenic effects of orexins are mainly mediated by the NPY system located in the ARC (Figure 1). Interestingly, immunohistochemical studies have demonstrated that orexin axons possessed synaptic interactions with NPY/AgRP neurons in the ARC and with NPY axons in the PVH (60). In harmony with this, OX-A within the PVH modulates spontaneous firing of glucose-sensitive neurons and promotes food intake via the NPY pathway (61). Furthermore, selective antagonists of NPY Y1 and NPY Y5 receptors centrally administrated partially reverse orexin-induced feeding stimulation (45, 62–64). Lastly, the central administration of OX-A stimulates NPY expression in the ARC, without affecting it in the DMH (40). Interestingly, AgRP, which is co-expressed with NPY in the ARC, was not modified upon OX-A treatment (40). Even if it may be argued that those central injections are given in a supra-physiological range, the involvement of alternative central mechanisms (i.e., NPY neurons) and the results obtained from *ox* null mice studies rule out the possibility of unspecific actions in terms of feeding control. In this sense, comparable results regarding feeding and alterations of NPY mRNA expression were observed in goldfish (65), suggesting a possible interaction between orexin and NPY systems to stimulate food intake (40, 65), which notably is evolutionary preserved, at least until the fish lineage. OX-A feeding effects have also been associated to other central mechanisms modulating food intake, such as endocannabinoids (66) CRH (45), urocortin, and melanocortins (67). In this regard, LHA orexin expression has been described to be higher in *Pomc* null mice, with a reversion following a central administration of α-MSH (68). As the orexin expression remained elevated in *pomc* null mice pair-fed (taking α-MSH-treated animals as a reference), this effect was independent of the primary actions of α-MSH on food intake. These data indicate that the elevation in orexin levels may be related to the hyperphagia observed in melanocortin deficient mice. To further support this evidence, that *ob/ob* mice show higher levels of OX-A in the ARC in concomitance with a reduction on *pomc* expression (35). Moreover, the OX-A-induced reduction of *pomc* mRNA expression and α-MSH production is reversed by administration of SB-334867 in obese mice (35).

Aside from their major role in the stimulation of food intake, orexins were also described to be involved in the sleep/wake cycling and pathology of narcolepsy (57–59). Numerous evidences—such as the loss of orexin containing neurons, mutations occurring in prepro-OX and OXRs, or reductions in cerebrospinal fluid (CSF) OX-A levels -, have linked orexins and narcolepsy in different species such as dogs, mice, rats or humans (20, 56–59, 69, 70). Apart from their implication in sleep/wake modulation, orexins are also involved in arousal regulation. Numerous anatomical evidences—orexin neuronal projections into PVH, locus coeruleus (LC) and dorsal raphe (71, 72) (main sites of productions of CRH, norepinephrine and serotonin) -, have confirmed this orexin/arousal regulation connection. Indeed, the central injections of orexins in rats induced different physiological behaviors, such as grooming [indicating a circadian dependence (73)], face washing, burrowing and searching (74–77). Altogether, these evidences have suggested that orexin feeding stimulation could rather be due to an increase of activity during the awake state (73) and to their stimulatory effect on arousal and vigilance, two features essential for normal feeding (74). Nevertheless, OX-A actions on feeding were described to be independent of arousal stimulation (20, 78).

## Nutrient Sensing by Orexins/Hypocretins

The orexin system is able to detect any changes in energy balance, such as fasting that leads to an increase of orexin mRNA and protein levels in rat hypothalamus (19, 37, 79, 80). In line with these findings, orexin neurons localized in the LHA can sense leptin and glucose levels. Actually, in orexin neurons, in an insulin-induced hypoglycaemia state, c-FOS (81) and hypothalamic prepro-OX mRNA (82, 83) expression are increased. Thus, a drastic decrease of glycaemia, detected by glucose-sensitive neurons (GSNs), could promote food intake. Interestingly, orexin axons have synaptic contacts with GSNs, and spontaneous GSNs firing rate is increased following OX-A release (61, 84–86). Accordingly, elevated blood glucose levels decrease orexin neurons firing rates, implying that orexins could be implicated in a negative feedback loop in order to counter any energetic variations (87). Moreover, it has also been described that orexin at a central level could bidirectionally modulate hepatic gluconeogenesis, engendering daily blood glucose oscillation (88–90). It was also described that aging could alter this regulation, being implicated in the circadian modulation of hepatic insulin. Indeed, the absence of orexin increases hepatic endoplasmic reticulum (ER) stress leading to decreased hepatic insulin sensitivity and altered gluconeogenic activity (90).

It was also proposed that leptin, a hormone released by the adipose tissue, could exert its appetite suppressant effect through the inhibition of orexin neurons activity (91). In this sense, it has been recently reported that OX neurons of *ob/ob* mice are innervated by less efficient and fewer excitatory synapses than wildtype mice. Moreover, in *ob/ob* mice, chronic absence of leptin induces a 2-AG mediated functional disinhibition of OX neurons, which would lead to increased OX production and therefore hyperphagia (92). Those things said, the interaction between leptin and orexins is complex. Indeed, controversial results are presented in the literature concerning the presence of leptin receptors on orexin neurons: while some are describing a colocalization (60, 93, 94), others were unable to do so (95, 96). Interestingly, the high levels of prepro-OX and OX1R mRNA encountered in rat hypothalamus under fasting conditions were decreased following an administration of leptin (19, 37). Furthermore, LHA orexin-A expression is reduced following leptin administration (97). In the same line of findings, it was demonstrated that in human plasma, the levels of OX-A were negatively correlated with the ones of leptin (35, 98). Despite these observations, low prepro-OX mRNA and high OXRs mRNA levels have been observed in the brains of obese *Zucker* rats (99, 100). Similar unexpected results were observed in *ob/ob* and *db/db* mice, in which low levels of prepro-OX mRNA in the LHA were detected, likely due to hyperglycemia (101). Recently, it has been proposed that leptin and orexin could act in a synergic manner in order to regulate energy sensing, particularly in the long term (102). In summary, regarding the close and complex interaction between orexin neurons and leptin signaling, further investigation would be needed to obtain an improved overview of all these mechanisms.

Ghrelin, an orexigenic hormone released by the gut, stimulates orexin neurons (103, 104). It was demonstrated that ghrelin could increase the rewarding value of palatable food through the stimulation of dopaminergic neurons located in the ventral tegmental area (VTA); this effect being inhibited using orexin antagonists (96, 105, 106). Despite these findings, it was demonstrated that the levels of prepro-OX mRNA were not modified after a treatment with ghrelin (107). Eventually, it has been suggested that feeding-related signals originating from the gut could modulate orexin signaling, via the vagus nerve and the NTS. Consequently, different stimuli, such as gastric expansion or glucose variations could act as feeding suppressing signals, having an important role in the regulation of orexin signaling (85, 108).

Recently, our group has demonstrated an interaction between the hypothalamic-pituitary axes and the central orexigenic action of OX-A. Hypophysectomized (HPX), adrenalectomized (ADX), gonadectomized (GNX; females and males), hypothyroid and GH-deficient dwarf rats were centrally injected with OX-A. Interestingly, we were able to show that the orexigenic effect of OX-A was completely maintained in ADX and GNX rats, slightly decreased in hypothyroid rats and entirely inhibited in hypophysectomized and dwarf rats (109). Remarkably, the loss of the OX-A effect on feeding was coupled with a blunted OX-A-induced increase of NPY or of its putative regulator, the transcription factor cAMP response-element binding protein (CREB), as well as its phosphorylated form pCREB, in the ARC of HPX and dwarf rats (109). All these results highlight the fact that the orexigenic effect induced by OX-A is dependent of the integrity of the GH axis (109). Moreover, our group has also demonstrated that OX-A inhibited GH secretion *in vivo* (110). Thus, this neuroendocrine feedback regulation could help to obtain a better understanding of orexin role in energy balance modulation and GH deficiency.

Recently, some growing evidences have linked orexin signaling to the amino acid (AA) sensing (111). Indeed, *Karnani and colleagues* have shown that nutritionally relevant mixtures of amino acids could stimulate orexin neurons both *in vitro* and *in vivo* (following peripheral and central administration). They have also proposed that this effect could be mediated by a dual mechanism involving the inhibition of K(ATP) channels and the activation of system-A amino acid transporters. Interestingly, they were also able to show that physiological concentrations of AAs inhibited the glucose responses of orexin neurons (111).

## Orexin/Hypocretins and Thermogenesis

The energetic metabolism relies on an accurate balance between energy intake and energy expenditure. Energy intake is mainly defined by the sum of caloric content of the ingested food and beverage. On the other side, energy expenditure is the sum of the thermic effect of food, locomotor activity and thermogenesis (112–114). Interestingly, the *obligatory thermogenesis*—defined as the heat produced by the metabolic rate—is enough to preserve the body temperature at adequate levels without involving any other thermoregulatory mechanisms. The temperature range in which the organism does not need to regulate its body temperature is called *thermoneutrality* (112–114). Temperatures below the *thermoneutrality* point lead to an immediate and quick response through the activation of heat saving mechanisms such as vasoconstriction or piloerection. However, this primary response only provides limited effects on maintaining body temperature. Thus, additional thermogenic mechanisms—referred to *facultative or adaptive thermogenesis*—are quickly initiated (112–114). These mechanisms are separated between *shivering and non-shivering facultative thermogenesis*. Shivering is an elementary response producing small amount of heat in cold-exposed organism (112–114). The evolutionary process allowed homoeothermic species to improve those mechanisms using metabolic machinery to generate heat in a more efficient way, named as *non-shivering facultative thermogenesis*. In mammals, including humans, the majority of *non-shivering facultative thermogenesis* is occurring in the brown adipose tissue (BAT) (112–114). BAT thermogenic capacities are mainly mediated by uncoupling protein 1 (UCP1), localized in the inner mitochondrial membrane. UCP1 dissociates the electron transport chain from ATP production by allowing the free movement of protons back across the mitochondrial membrane, increasing the energy dissipation as heat (112–114).

Since a long time, it is well known that the stimulation of LHA activates BAT (1, 6, 7, 26, 115, 116). In the same line of findings, numerous studies, including pharmacological experiments and genetic modified mouse models, highlighted the fact that orexins promoted energy expenditure. Indeed, OX-A centrally administrated in mice under fasting increased the metabolic rate (117, 118) and induces a hyperthermic response (119). *Ox* null mice developed a hypometabolic phenotype (57) and more sensitivity to cold exposure (120). Conversely, OX-A centrally administrated has also been described to induce hypothermic effects, through a NPY-dependent mechanism (121, 122). Intriguingly, another study has reported that OX-B could induce hyperthermic effects (122). Interestingly, these effects can be reproduced by an OX-B administration into the diagonal band of Broca (123). The exact significance of the orexin induced thermogenic effect is still questionable, but it has been proposed that it could be an adaptive response to stress (124) or to cold exposure (120).

Molecular studies have revealed that orexins could modulate both locomotor activity and BAT thermogenesis to induce their effects on energy expenditure (125–130). Recently, it has been reported that orexins were essential for BAT development, differentiation, and function (125). As observed in *ox* null mice, the lack of orexin deregulates energy balance (120, 125). This evidence is also supported by *in vitro* studies that have revealed a direct implication of orexin on new brown adipocytes differentiation (125, 129). Morphological analysis has also shown that orexin neurons were implicated in the modulation of BAT thermogenesis through the SNS (Figure 1). Accordingly, (i) the central administration of OX-A or OX-B, (ii) the stimulation of orexin neurons in the LHA or (iii) the injection of OX-A specifically into the VMH or in the RPa promoted BAT thermogenesis (116, 126, 128, 131–133) while (iv) rats with ataxin-3 mediated ablation of orexin neurons showed reduced BAT thermogenesis (134, 135). Even if the mechanisms underlying these effects remain unclear, it was recently reported that AMP-activated protein kinase (AMPK), a cellular energy sensor (5, 136, 137), and endoplasmic reticulum stress (ER stress), a cellular process that is triggered by a variety of conditions that disturb folding of proteins in the ER (138–140), were the main modulators of BAT thermogenesis in the VMH (Figure 1) (5, 120, 141–155). Thus, it seems essential to pursue the investigation to understand how orexins, AMPK and ER stress could interact to modulate BAT thermogenesis in the hypothalamus. In this sense, recent data from our group have demonstrated that the thermogenic effect of the bone morphogenetic protein 8B (BMP8B, a thermogenic factor initially involved in bone morphogenesis) (120, 143) on BAT (as well as the browning of white fat) is mediated by the inhibition of AMPK in the VMH and by the subsequent increase in OX signaling via the OX1R. Accordingly, the thermogenic effect of BMP8B is totally absent in *ox* null mice implicating glutamatergic signaling, indicating a physiological role of orexins in this regard (120). To understand whether these effects could also involve hypothalamic ER stress would require further investigations, however considering that the link AMPK-ceramide-induced-ER stress has already been demonstrated for thyroid hormones (153), it is tempting to speculate a possible role on orexin actions. Additional investigations would be needed to further understand the role of orexins on BAT function and whether this action could have evolutionary implications related to adaptations to environmental temperature. Therefore, it will be demanding to investigate the effect of orexins and its postulated downstream and upstream regulators (for example AMPK) at different temperatures, such as cold exposure and thermoneutral conditions.

Recently, it has also been reported that orexin could have a protective role in aging-associated impaired thermogenesis (130). It is well known that aging induces an increase in fat mass, however the underlying mechanisms are still unknown. Numerous evidences have shown that aging was associated with impaired differentiation of BAT, morphologic malformations and thermogenic dysfunctions in rodents (130). This loss of function can be explained by the fact that in aged mice, the interscapular brown fat region is invaded by white-like adipocytes (130). Additionally, old mice are unable to mobilize brown adipocyte intracellular energetic reserves leading to an impaired regulation of basal thermogenesis. Interestingly, OX-A administration reverses these effects; while they were described to be potentialized in mouse with ablated OX neurons (130). Further investigations are needed to evaluate whether the orexin system could be a potential target to reverse the fat mass increase associated with aging. In this regard, considering the involvement of orexin in pathophysiology in humans (i.e., narcolepsy), and the presence of brown/browned fat in humans (149, 156–159), another important question to answer is whether orexins may play a role in the modulation of BAT in humans. Current evidence has demonstrated that contrary to rodents, OX-A treatment alone or in combination with an adrenergic stimulus did neither enhance thermogenesis nor its related transcriptional program in a human *in vitro* model of brown adipocytes or adipose tissue explants (160). These results are in keeping with data demonstrating that although narcolepsy patients show abnormal fat distribution (161), they do not display differences in the amount of supraclavicular BAT (161). Moreover, it has been shown that BAT is perfectly functional after cold exposure in patients with narcolepsy (162). Overall, this evidence indicates that the role of orexins on BAT activity in human is at least controversial. Further work will be required for a deeper investigation that will allow to address whether the effects of orexins on BAT are a specific phenomenon for rodents or, alternatively, a pathway that might be susceptible of therapeutic intervention in humans.

One interesting point is that orexins are primarily stimulated by fasting and starvation, a situation in which animals display hypothermia, reduced oxygen consumption and blunted thermogenic responses. Overall, these responses could seem contradictory with the physiological role of orexins as stimulators of BAT activity. However, the role of orexins in each scenario is totally dependent of other factors such as (i) the hormonal milieu (for example, ghrelin, leptin and thyroid hormone levels), (ii) the upstream and downstream hypothalamic molecular pathways and (iii) the hypothalamic nucleus specificity. In a fasting scenario, considering the role of orexin neurons as sensors of the outer and inner environment to reach a state of vigilance and wakefulness for seeking food, an increase of the orexigenic tone could be expected (22, 25). The mechanisms leading to this increase of orexin levels are numerous, including ghrelin and leptin which are essential as they play opposite role on orexin neurons (an increase in ghrelin levels and a decrease in leptin ones is observed during fasting) (37, 87). Ghrelin activates isolated orexin neurons inducing their depolarization and an increase in firing frequency (87). In contrast, leptin, a strong anorectic factor, robustly inhibits orexin neurons, causing hyperpolarization and decreasing the firing rate (87). This is the reason why in mice lacking orexin neurons, the fasting-induced arousal is impaired (87). On the other hand, conclusive evidence exists demonstrating that under normal feeding status, orexin neurons activate BAT through a mechanism involving elevated AMPK activity and expression in the VMH (5, 120). This blunted response in starvation is not so surprising as it reminds similar phenomena observed with thyroid hormones, in which, their effects on thermogenesis are also blunted. Under these circumstances, there is a change in the set point governing the entire hypothalamic-pituitary-thyroid axis to save as much energy as possible to ensure survival (114). In other words, under starvation conditions, the orexin system is activated allowing food-seeking, while the signaling mechanisms implicated in increasing thermogenesis are blunted to preserve energy. The exact mechanisms governing this later phenomenon remain unknown but it clearly merits to be pursued. However, some hypotheses can be speculated. During fasting, increased orexin levels were shown to induce feeding by stimulating NPY neurons in the ARC (40), which are also stimulated by the fasting-induced increase in ghrelin and the fasting-induced decrease in leptin (107). NPY neurons are known to inhibit thyrotropin-releasing hormone (TRH) neurons in the PVH, leading to decreased thyroid-stimulating hormone (TSH) release from the pituitary and subsequently reduced circulating thyroid hormones and reduced thermogenesis (114, 141, 153, 163, 164). Notably, as a result of the decreased thyroid hormone tone, most of that effect could be mediated by increased AMPK in the VMH (acting upstream of orexin) (114, 120, 141, 153, 163, 164). Therefore, the interplay between hormonal factors (ghrelin, leptin, thyroid hormones), neuropeptides (NPY and orexin) and energy sensors (AMPK) could be implicated into this complex regulation. Another possible scenario might be an adaptation to cold temperatures, a situation in which both thyroid hormones and orexins are known to be elevated (114, 134, 135), which would lead to (i) increased BAT thermogenesis (induced by both thyroid hormones and orexins) and (ii) increased feeding (again, induced by both thyroid hormones and orexins) to cope the higher metabolic demands of thermogenesis. Similar thoughts are described for BMP8B, which induces thermogenesis, acting on orexin neurons in the LHA and AMPK in the VMH (120). Therefore, the pleiotropic role of orexins, as well as the redundancy of the hypothalamic networks modulating whole body energy homeostasis, are determining the response in each physiological condition.

## Concluding Remarks

All throughout this review was described the implication of the orexin and hypocretin systems in the control of energy balance (Figure 1). Even if their roles in the regulation of the two components of the energy balance, namely feeding and energy expenditure, are well characterized, their involvement in the development in obese disorders remain unclear and debated. Indeed, orexin, centrally administrated, did not induced any body weight modifications (42) as well as prepro-OX mRNA levels remained unchanged in diet-induced and genetic models of obesity (165, 166). However, as mentioned above, the major role of orexins in the regulation of energy balance is undeniable. However, due to their pleiotropic characteristics, considering them as potential therapeutic targets in an obesity driven context appears as limited (167). To overcome these actual limits, a better understanding of orexin interaction with other known, or even unknown, systems involved in energy balance control would be necessary.

## Author Contributions

All authors listed have made a substantial, direct and intellectual contribution to the work, and approved it for publication.

### Conflict of Interest

The authors declare that the research was conducted in the absence of any commercial or financial relationships that could be construed as a potential conflict of interest.
